# Transcorneal Electrical Stimulation Induces Long-Lasting Enhancement of Brain Functional and Directional Connectivity in Retinal Degeneration Mice

**DOI:** 10.3389/fncel.2022.785199

**Published:** 2022-02-07

**Authors:** Stephen K. Agadagba, Abdelrahman B. M. Eldaly, Leanne Lai Hang Chan

**Affiliations:** ^1^Department of Electrical Engineering, City University of Hong Kong, Kowloon, Hong Kong SAR, China; ^2^Electrical Engineering Department, Faculty of Engineering, Minia University, Minia, Egypt

**Keywords:** electrocorticography (ECoG), retinal degeneration, transcorneal electrical stimulation, theta-gamma coupling, brain coherence, brain connectivity analysis

## Abstract

To investigate neuromodulation of functional and directional connectivity features in both visual and non-visual brain cortices after short-term and long-term retinal electrical stimulation in retinal degeneration mice. We performed spontaneous electrocorticography (ECoG) in retinal degeneration (rd) mice following prolonged transcorneal electrical stimulation (pTES) at varying currents (400, 500 and 600 μA) and different time points (transient or day 1 post-stimulation, 1-week post-stimulation and 2-weeks post-stimulation). We also set up a sham control group of rd mice which did not receive any electrical stimulation. Subsequently we analyzed alterations in cross-frequency coupling (CFC), coherence and directional connectivity of the primary visual cortex and the prefrontal cortex. It was observed that the sham control group did not display any significant changes in brain connectivity across all stages of electrical stimulation. For the stimulated groups, we observed that transient electrical stimulation of the retina did not significantly alter brain coherence and connectivity. However, for 1-week post-stimulation, we identified enhanced increase in theta-gamma CFC. Meanwhile, enhanced coherence and directional connectivity appeared predominantly in theta, alpha and beta oscillations. These alterations occurred in both visual and non-visual brain regions and were dependent on the current amplitude of stimulation. Interestingly, 2-weeks post-stimulation demonstrated long-lasting enhancement in network coherence and connectivity patterns at the level of cross-oscillatory interaction, functional connectivity and directional inter-regional communication between the primary visual cortex and prefrontal cortex. Application of electrical stimulation to the retina evidently neuromodulates brain coherence and connectivity of visual and non-visual cortices in retinal degeneration mice and the observed alterations are largely maintained. pTES holds strong possibility of modulating higher cortical functions including pathways of cognition, awareness, emotion and memory.

## Introduction

The human brain is a dynamic organ that produces large scale coordinated electrophysiological activities in billions of neurons. These activities are represented as repetitive patterns of brain oscillations. Brain oscillations are capable of interacting with each other at different frequencies; a concept called cross-frequency coupling (CFC). This rhythmic interaction between synchronous neural oscillations is a known fingerprint of functional coupling. CFC has been reported to occur in various brain regions including deep brain structures such as the hippocampus (Michaels et al., 2018), amygdala (Zheng et al., 2019), cortical areas of the prefrontal cortex (Helfrich et al., 2017), motor cortex (De Hemptinne et al., 2013) and visual cortex (Sokoliuk and VanRullen, 2012). Notwithstanding the area of occurrence, CFC often features an interaction where the phase of low frequency oscillations such as theta (5–10 Hz) or alpha (10–15 Hz) has been demonstrated to couple or modulate the amplitude of high frequency gamma oscillations (30–100 Hz) (Zhang et al., 2019). This phase amplitude coupling (PAC) of the associated brain rhythms has been severally proven to play important roles in higher cognitive functions of the brain including visual functions (Sokoliuk and VanRullen, 2012), working memory (Lega et al., 2014) and behavioral functions (Ohki et al., 2020) amongst others.

When more than one brain region is considered, brain coherence may be established owing to a high degree of association or similarity in neural activities between the associated brain regions. Apart from interpreting only the similarity in neural activities between brain sites, the directional connectivity between the brain regions must also be taken into account. For example, visual recognition of letters in the human brain specifically involves connectivity networks arising from the occipital lobe in the brain posterior to the anterior regions of the brain (Garagnani et al., 2017). Similar to CFC, both coherence and directional connectivity measures are also used to infer a functional relationship between different regions of the brain. In essence, coherence and directional connectivity between brain regions are of paramount importance in establishing coordinated network activities for physiological brain functionality.

Evidence from scientific research has shown that the pathogenesis of neurodegeneration is linked to progressive and wide spread changes in neural networks of the brain (McMackin et al., 2019). Furthermore, it has been demonstrated that drastically reduced brain coherence and connectivity are key features of several neurological and neurodegenerative disorders such as schizophrenia, Parkinson’s disease, epilepsy, autism and Alzheimer’s disease (Nakamura et al., 2017; Bazzigaluppi et al., 2018; Salimpour and Anderson, 2019). Consequently, electrical stimulation strategies have been studied to modulate neural oscillations and ultimately re-adjust dysfunctional brain connectivity networks (Salimpour and Anderson, 2019). Indeed, it has been reported that transcranial alternating current (Vossen et al., 2015) and transcranial direct current (Jamil et al., 2017) stimulation paradigm modulates percepts in the brain and the aftereffects outlast the electrical stimulation period. At this juncture, it is crucial to mention that the neural information that originates from the retina is integrated by systems that are in active communication with one another including motor, cognitive and emotional systems (Mulckhuyse, 2018; Song et al., 2019; Costumero et al., 2020). With research progress in retinal signaling pathways, it is now becoming clearer that multiple coherence and connectivity pathways exist between the retina and other non-visual brain regions such as the prefrontal cortex, motor cortex, hippocampus and the amygdala (Preston and Eichenbaum, 2013; Turk-Browne, 2019). A typical example is the fear-emotional system which has been reported to involve a conglomeration of neural inputs from the retina, the prefrontal cortex, the hippocampus, the amygdala and the motor cortex (McFadyen et al., 2019). These inputs are both excitatory and inhibitory and occur *via* feedforward and feedback mechanisms in the associated brain regions (Suzuki and Amaral, 1994).

Similar to the aforementioned diseases, retinitis pigmentosa is also a neurodegeneration disease that involves progressive degeneration of light sensitive photoreceptor retina neurons and ultimately leads to vision loss or blindness. It has been reported that despite retinal degeneration, the retinotopic map is largely preserved (Xie et al., 2010). Consequently, several approaches are being studied for vision restoration in RP patients including retina electrical stimulation *via* invasive and non-invasive routes (Chow, 2013; Rahmatnejad et al., 2016; Beyeler et al., 2019; Chenais et al., 2021). Arising from this and from the clear evidence that the retina of the eye forms a neural-link with the brain (Murphy et al., 2016; Varadarajan and Huberman, 2018), it is therefore crucial to understand the short-term and long-term implication of retina electrical stimulation on different brain regions. Currently, there is no systematic study about the short-term and long-term neurophysiological effects of prolonged retinal stimulation in visual and non-visual regions of the brain. Furthermore, retinal prostheses are designed to treat diseases of the outer retina, such as Age-related Macular Degeneration (AMD) and Retinitis Pigmentosa (RP), which blind hundreds of thousands each year. These visual devices are implanted to electrically stimulate the retina circuitry with the aim of providing partial or complete vision restoration. Nonetheless, because retinal prostheses need to be turned on continuously to aid the daily life activities of the implant recipients, the long-term use of these devices is hypothesized to unavoidably trigger neuromodulation in both visual and non-visual regions of the brain. In our previous studies, we demonstrated that repetitive prolonged transcorneal electrical stimulation (pTES) is able to modulate the resting state brain activity of retinal degeneration (rd) mice in a frequency (Agadagba and Chan, 2020; Agadagba et al., 2020) and pulse duration-dependent manner (Agadagba and Chan, 2021). We also observed that the awake brain was more responsive to the effect of retinal electrical stimulation in comparison to the anesthetized brain (Agadagba et al., 2020). This finding about brain states has also been corroborated by other authors and it has been linked to variations in cortical processing between the two brain states (Keller et al., 2012; Durand et al., 2016). From the foregoing, in our present study we have exclusively used the retinal degeneration (rd) mice model of the human blinding disease retinitis pigmentosa to decipher the effect of a prolonged electrical stimulation of the retina from a brain-wide perspective.

Brain coherence and connectivity indices have been previously studied in the resting brain state by techniques that analyze continuous electrophysiological signals such as local field potential (LFP), electrocorticogram (ECoG) and electroencephalogram (EEG) (Young and Eggermont, 2009). Specifically, ECoG is unique in approach and has high spatio-temporal resolution which allows tracking of neural oscillatory coherence and connectivity that occurs in the CNS. In the present study we therefore used resting state ECoG to investigate neuromodulation of functional and directional brain connectivity in both visual and non-visual cortices of rd mice after short-term stimulation (transient or 1-day post-stimulation) and long-term stimulation (1-week post-stimulation and 2-weeks post-stimulation), respectively.

## Materials and Methods

### Animals and Surgery

**FIGURE 1:**
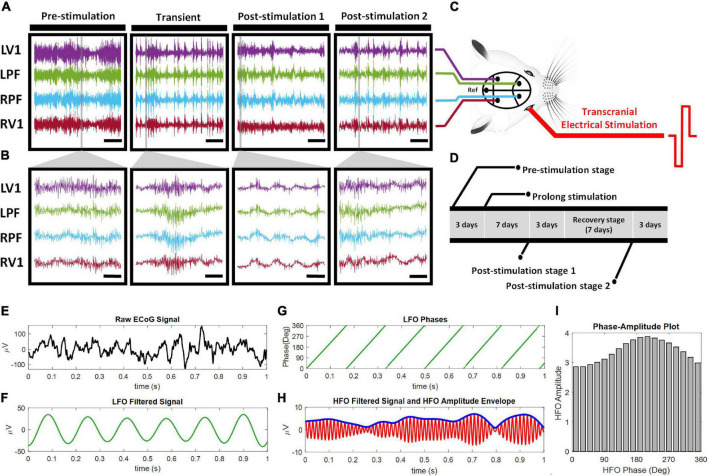
ECoG Experimental design and steps taken to compute cross-frequency phase-amplitude coupling. **(A,B)** Represent ECoG signal traces from one rd10 mouse. ECoG signal was recorded from the four brain sites: the left primary visual cortex (LV1), the left prefrontal cortex (LPF), the right prefrontal cortex (RPF) and the right primary visual cortex (RV1) across four stages of stimulation (pre-stimulation, transient, post-stimulation 1 and post-stimulation 2) **(A)** scale bar, 5 s, **(B)** Scale bar, 100 ms. **(C)** Pictorial representation of the ECoG recording positions in the mouse brain and biphasic transcorneal electrical stimulation applied to the right eye. Ref: reference electrode position. **(D)** Timeline of experimental design. Transient stage ECoG recording was performed on the first day of the 7 days prolonged stimulation. The raw ECoG signal **(E)** was filtered into phase of LFO **(F)** and amplitude (thin line) of HFO **(G)**. The time series of LFO phase and amplitude envelope of HFO (thick line) was computed from the respective filtered ECoG signals *via* Hilbert transformation **(H)**. The phase amplitude plot **(I)** was constructed and used to calculate the mean distribution of amplitudes over the phase bins.

In all experiments, retinal degeneration 10 (rd10) mice were used as animal models of human RP. The mice were bred in Laboratory Animal Research Unit (LARU) at City University of Hong Kong. All procedures for animal handling and animal experimentation were reviewed and approved by the Animal Research Ethics Sub-Committee at City University of Hong Kong (A-0249) and were carried out in compliance with the Animals (Control of Experiments) Ordinance at Department of Health, Hong Kong SAR (20–162 in DH/HT&A/8/2/5 Pt.3). A total of forty rd10 mice (20 males and 20 females) of post-natal age P60–P90 were used in this study. All surgical steps were same as described in our previous research (Agadagba et al., 2019). To summarize the surgical procedures, rd10 mice were first injected with ketamine-xylazine anesthesia (ketamine: 100 mg/kg, Xylazine: 10 mg/kg), followed by administration of mixed anesthesia [isoflurane (1.5%) and medical oxygen (0.4%)] to maintain the sleep state during the surgery. Each mouse was head fixed on a stereotaxic (Stoelting, CA, United States) and after surgical craniotomy, four stainless steel bone-screw recording electrodes of shaft diameter 2.4 mm were implanted subdurally (Model# EDP81912; Decorah LLC, GEC, United States). For the visual region, we implanted the recording electrode bilaterally over the primary visual cortex (Anterior—Posterior: –3.5 mm, Midline: 2.5 mm) (Figures 1A–C). For the non-visual region, we implanted the recording electrode bilaterally over the general region of the frontal cortex (Anterior—Posterior: 2 mm, Midline: 1 mm) in the vicinity of the medial prefrontal cortex (mPFC) and the frontal association region (Figures 1A–C). Henceforth, we refer to this region as the prefrontal cortex of the rd10 animals. It is important to state here that in the present study we used the term “non-visual region” exclusively in the context that the prefrontal cortex although involved in image interpretation, however, it does not receive direct visual signals from the receptive fields of the retinal neurons compared to the primary visual cortex. Following implantation in the regions of interest, the reference bone-screw was positioned in the cerebellar region (Figure 1C). Heart rate of all mice was carefully monitored and their body temperature was kept constant with a heat pad (Model # TP702; Gaymar Industries, Inc., NY, United States) at 38°C. To prevent mice eyes from drying out during surgery and recording sessions, lubrithal eye gel was applied. After surgery, all animals were allowed to recover for 1 week (7 days) before further experimentation.

### Retinal Stimulation

Following anesthesia administration with mixture of isoflurane (1.5%) and medical oxygen (0.4%), all mice were mounted on the stereotaxic (Stoelting, CA, United States) in preparation for retinal stimulation. Retinal stimulation of rd10 mice was done by transcorneal electrical stimulation (TES). Similar to our previous studies (Agadagba et al., 2019, 2020; Agadagba and Chan, 2021), we used TES in the present study because the mouse eye is small in size (approximately 3 mm in diameter) thus no commercially available retinal implants for stimulation of the mouse eye. TES therefore provided a readily applicable non-invasive stimulation approach for the mouse eye with no known post-surgical complications. The procedure of TES was similar to our previous studies (Agadagba et al., 2020; Agadagba and Chan, 2021). Briefly, a silver wire electrode used as the stimulating electrode (impedance: 0.143 kΩ at 1 kHz) was initially connected at one end to an electrical pulse generator (Multichannel Systems STG stimulator, Model # 4004; Baden-Württemberg, Germany). The other end of the silver wire electrode was positioned on the right cornea of rd10 mice (Figure 1C). A needle reference electrode was positioned subcutaneously in close proximity to the right eye. TES was applied for a prolonged period of 30 min (hence we dubbed the stimulation as “pTES”) and repeated over the course of 7 days. Both short-term and long-term effects of pTES were investigated as described in section “Electrocorticography Signal Acquisition.”

Three test groups (with ten rd10 mice per group) were set up. Each test group was injected with charge balanced biphasic square-wave pulses of varied current intensity (400, 500, and 600 μA, respectively), at 10 Hz stimulation frequency and 2 ms/phase pulse width. In this regard, the charge injected per phase for each test group was 0.8 μC, 1 μC and 1.2 μC, respectively. A sham control group of rd10 mice (*n* = 10) was also set up and treated in the same manner as the test groups but without retinal electrical stimulation. All mice were closely monitored during and after all experimental procedures. All mice showed no sign of discomfort or emotional stress.

### Electrocorticography Signal Acquisition

ECoG was used to record spontaneous electrophysiological activity over the cortical surface of awake rd10 mice. The ECoG signal was acquired and amplified by connecting an active transfer cable from the recording electrode to a data acquisition system (Model # Micro 1401-3; Cambridge Electronic Design, United Kingdom). During the process of signal acquisition, ECoG signal was sampled at 5 kHz from 0.3 to 300 Hz. A signal gain function of 1 K and 50 Hz notch filter was applied (Model # 3600; A-M Systems, Washington, United States) for signal amplification and noise cancelation. ECoG signal recording time was 10 min in duration. In order to investigate the short-term and long-term effects of pTES, we acquired data from ECoG recording for four stimulation stages namely pre-stimulation, transient stage, post-stimulation stage 1 and post-stimulation stage 2 (Figures 1A,B). For the short-term effects of pTES, transient stage ECoG activity was recorded only on day 1 of pTES while for the long-term effects of pTES, post-stimulation stage 1 ECoG activity was recorded 1-week after the start of pTES and post-stimulation stage 2 was recorded at 2-weeks after the start of the 7-days pTES (from day 17). Hence, we also refer to transient stage as day-1 post-stimulation, post-stimulation stage 1 as 1-week post-stimulation and post-stimulation stage 2 as 2-weeks post-stimulation (Figures 1A,B,D).

### Signal Pre-processing and Signal Analysis

ECoG signal from this study was pre-processed by de-noising and down-sampling. As mentioned previously during online data sampling, 50 Hz notch filter was applied to the data amplifier system to remove interfering 50 Hz and its super-harmonics originating from the alternating current (AC) line. Following data acquisition, a fair amount of 50 Hz noise observed was eliminated by an infinite impulse response comb (IIR-comb) filter in MATLAB algorithm (MathWorks, Inc., R2018b, Natick, Massachusetts, United States). There were approximately 3 × 10^6^ data points obtained from each recording electrode channel which was very large and had the potential to increase computation time. To solve this problem and reduce computation time during data analysis, the ECoG signal was down-sampled from 5 to 1 kHz with MATLAB algorithm (MathWorks, Inc., R2018b, Natick, Massachusetts, United States).

### Analysis of Cross-Frequency Coupling

CFC between low frequency oscillations (LFOs) and high frequency oscillations (HFOs) was analyzed for the left (contralateral) prefrontal cortex and the left (contralateral) primary visual cortex channels by PAC method (Figures 1E–I). Estimation of modulation index (*MI*) was used to quantify the degree of coupling between associated brain oscillations based on Kullback-Leibler method (Tort et al., 2010). Briefly, linear finite impulse response (FIR) filter was used to filter the raw data into LFOs (1–29 Hz) for phase and HFOs (10–295 Hz) for amplitude. The phases and amplitudes of the LFOs and HFOs, respectively, were extracted by Hilbert transform from the time series of each filtered signal, followed by coupling into one composite. Next, the phases were binned into eighteen intervals (from 0° to 360° with step 20°).

The mean of the amplitude envelope over each phase bin was calculated to quantify amplitude distribution over phase bins. *MI* was computed by normalizing *H* (the entropy measure of the normalized amplitude distribution over phase bins) *via* the maximum achievable entropy value; *H*_max_ = log(*N*), where N equals to the number of phase bins (equation 1).


(1) $$M\,I=\left(H_{m\,a\,x}-H\right)/H_{m\,a\,x}$$

To test the statistical significance of the *MI* values, a distribution of 50 surrogate *MI* values was created by randomly shuffling the composite time series of high-frequency amplitude envelope and phases of a low-frequency signal after segmenting equally into 20 blocks. Assuming the surrogate *MI* values are normally distributed, the *MI* value of the original signal was considered significant depending on whether it reached the top 5% of this surrogate data distribution, otherwise it was ignored and replaced with zero. Thus, in comodulogram plots, any *MI* value greater than zero was statistically significant. For a particular channel, the averaged *MI* was calculated for all trials in each group. The comodulogram plots were obtained by representing the average *MI* values of multiple phase and pairs of amplitude frequency, computed in steps of 1 Hz with 2 Hz phase bandwidths and in steps of 5 Hz with 10 Hz amplitude bandwidths.

### Analysis of Coherence

The coherence between ECoG channels of the left prefrontal cortex and the left primary visual cortex was estimated by using the *mscohere.m* function in MATLAB (MathWorks, Inc., R2018b, Natick, Massachusetts, United States). Specifically, this gives the magnitude squared coherence *C*_*xy*_ (*f*). The magnitude squared coherence is a coherence estimate of two signals *x* and *y* based on their frequencies. *C*_*xy*_ (*f*) uses Welch’s averaged modified periodogram to assess the degree of association between input signals *x* and *y*.


(2) $$C_{x\,y}\,\left(f\right)=\frac{{\left|P_{x\,y}\,\left(f\right)\right|}^{2}}{P_{x\,x}\,\left(f\right)\,P_{y\,y}\,\left(f\right)},0\leq C_{x\,y}\,\left(f\right)\leq 1,$$

In equation 2 above, *P_*xx*_ and P_*yy*_* represent the power spectral density of the signals *x* and *y*, respectively; *P*_*xy*_ represents the cross-power spectrum spectral density. For each stimulation group in the present study, the mean coherence among a pair of two EEG channels (left prefrontal cortex and the left primary visual cortex) was calculated. Over the whole ECoG recording, ECoG signal was segmented into epochs of 2 s with 1 s overlap. Over the entire frequency range (0.5 Hz–300 Hz), *C*_*xy*_ was computed at each frequency bin and epoch for each mouse. Subsequently, the mean coherence and standard error of the mean (SEM) were then computed for all mice across the four stages (pre-stimulation, transient, post-stimulation stage 1 and post-stimulation stage 2).

### Analysis of Directional Connectivity

The directional connectivity between the ECoG channels of the left prefrontal cortex and left primary visual cortex was assessed by normalized symbolic transfer entropy (NSTE), which is a non-linear method for estimating directional functional connectivity on the basis of information theory. STE quantifies the dominating direction of information flow between time series from structurally or functionally coupled systems by estimating the amount of information between the future of the target signal and the past of the source signal given the knowledge delivered from the history of the target signal in the model, which is described as follows:


$$S\,T\,E_{X\to Y}=I\,\left(Y^{F};X^{P}|Y^{P}\right)$$

(3) $$=H\,\left(Y^{F}|Y^{P}\right)-H\,\left(Y^{F}|X^{P},Y^{P}\right),$$

where, *X*^P^*, Y*^P^**, and *Y^F^* represent the past of source and target signals; and the future of the target signal, respectively. *H*(*Y^F^*|*Y^P^*) is the entropy of the process *Y*^F^**, that is dependent on its past. In the present study, EEGlab version 15 (Swartz Center for Computational Neuroscience, CA) and EEGapp (BIAPT lab, McGill University) toolboxes were integrated with MATLAB (MathWorks, Inc., R2018b, Natick, Massachusetts, United States) in order to estimate the NSTE. Briefly, the ECoG signal was first filtered into eight frequency bands (delta, theta, alpha, beta, low gamma, medium-gamma, high-gamma and ultra-gamma) and then segmented the filtered data into 10-s-long ECoG epochs without overlapping. The potential bias of STE was eliminated with a shuffled data by subtracting the original STE and then dividing by the entropy of the target signal, *H*(*Y^F^*|*Y^P^*) (equation 4).


(4) $${\text{NSTE}}_{X\to Y}=\frac{{\text{STE}}_{X\to Y}-{\text{STE}}_{X\to Y}^{\text{Shuffled}}}{H\,\left(Y^{F}|Y^{P}\right)}\in \left[0,1\right],$$

The feedforward (FF) NSTE and feedback (FB) NSTE between the left prefrontal cortex and the left primary visual cortex was sequentially computed in each epoch for the 10 min recording duration. The directional connectivity between the left prefrontal cortex and the left primary visual cortex was defined by the average NSTE in each stimulation group across the four stimulation stages.

### Statistical Analysis

All statistical analyses were performed by using the software Origin (Pro) (Version 2015, Origin Lab Corporation, Northampton, MA, United States). To test the statistical significance of the change in PAC, coherence and NSTE across the four stages of stimulation, one-way ANOVA and Tukey’s multi-comparison tests were carried out for the eight frequency bands. The adjusted *P*-values by Tukey’s correction was *P < 0.025.

## Results

### Medium-Gamma Oscillations Are Coupled to Theta Waves in the Primary Visual Cortex Following Retinal Electrical Stimulation

**FIGURE 2:**
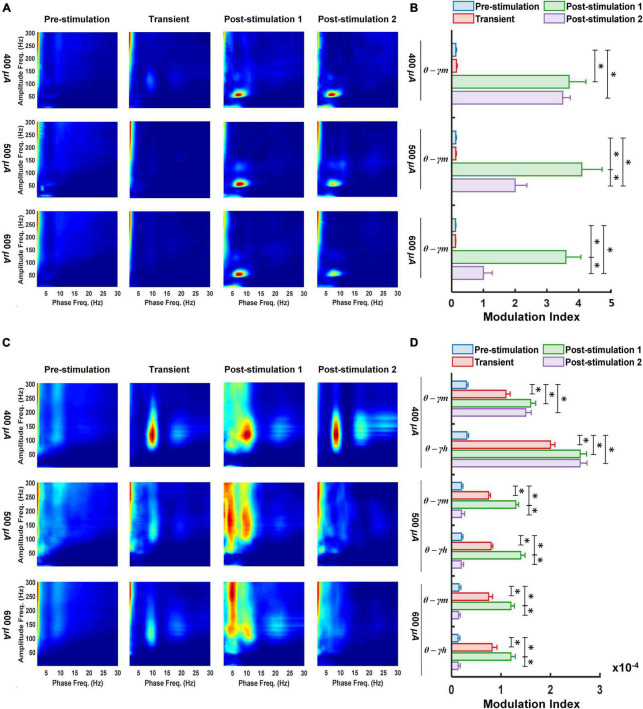
Following retinal electrical stimulation theta waves modulates medium-gamma oscillations in the left primary visual cortex **(A,B)** while modulating both medium-gamma and high-gamma oscillations in the left prefrontal cortex **(C,D)**. **(A)** Phase-amplitude comodulograms was computed for ECoG signals from 400 μA stimulation (*n* = 6), 500 μA stimulation (*n* = 6), 600 μA stimulation (*n* = 6) during pre-stimulation (baseline), transient stimulation, post-stimulation stage 1 and post-stimulation stage 2. **(B)** Mean *MI* values for phases of slow theta waves (5.5–10 Hz) and amplitudes of fast medium-gamma oscillations (60–115 Hz) in the left primary visual cortex. **(C)** Phase-amplitude comodulograms computed for ECoG signals from 400 μA stimulation (*n* = 6), 500 μA stimulation (*n* = 6), 600 μA stimulation (*n* = 6) during pre-stimulation (baseline), transient stimulation, post-stimulation stage 1 and post-stimulation stage 2. **(D)** Mean *MI* values for phases of slow theta waves (5.5–10 Hz) and amplitudes of fast medium-gamma (60–115 Hz) and high-gamma oscillations (125–175 Hz) in the left prefrontal cortex. Twelve types of cross-frequency coupling were investigated between the three phase frequency bands [delta (0.5–5 Hz), theta (5–10 Hz) and alpha (10–15 Hz)], and four amplitude frequency bands [low gamma (30–55 Hz), medium-gamma (60–115 Hz), high-gamma (125–175 Hz), and ultra-gamma (185–300 Hz)]. *θ—γm*, Theta medium-gamma coupling; *θ—γh*, Theta high-gamma coupling. * Significant (*P* < 0.025); Error bar denotes SEM.

It has been demonstrated that CFC between the phases of LFOs and the amplitudes of HFOs play important roles in visual-related activities (Spaak et al., 2012). Thus, in the left primary visual cortex we analyzed the effect of varying the stimulation current (400, 500, and 600 μA) on the coupling characteristics between the phase of delta, theta and alpha waves and the amplitude of a range of gamma oscillations (low gamma γ_*l*_, medium gamma *γ_*m*_*, high gamma *γ_*h*_* and ultra-gamma *γ_*u*_)*. The results obtained at each stage of pTES (transient, post-stimulation 1 and post-stimulation 2) were compared with the corresponding baseline (pre-stimulation) PAC results in rd10 mice. As shown in Figures 2A,B, for the transient stage all stimulation groups (400, 500, and 600 μA) yielded no significant change (*P* = 0.778, 0.997, and 0.918) in PAC of all assessed LFOs and HFOs compared to the corresponding pre-stimulation PAC. However, for 1-week post-stimulation (post-stimulation stage 1), all stimulation groups (400, 500, and 600 μA) displayed prominent increase (*P* = 0.009, 0.004, and 0.003) in theta medium-gamma PAC compared with the theta-medium gamma PAC at the pre-stimulation stage (Figures 2A,B).

Two weeks after the start of the 7-days pTES (2-weeks post-stimulation or post-stimulation stage 2), there was maintained elevated increase in theta-medium gamma PAC of rd10 mice stimulated with 400 μA (*P* = 0.010), 500 μA (*P* = 0.011), and 600 μA (*P* = 0.011), respectively, compared with theta-medium gamma PAC at the pre-stimulation stage (Figures 2A,B). Despite, the long-lasting enhancement in theta-medium gamma PAC among all stimulation groups, 400 μA stimulation appeared to elicit a stronger long-lasting enhancement in theta medium-gamma PAC compared with the 500 and 600 μA stimulations, respectively. This was evident because stimulation with 400 μA showed no significant change (*P* = 0.107) in the enhanced theta-medium gamma PAC between post-stimulation stage 1 and post-stimulation stage 2 compared to the significant change that occurred in the theta-medium gamma PAC between post-stimulation stage 1 and post-stimulation stage 2 with both 500 μA (*P* = 0.019) and 600 μA (*P* = 0.019) stimulations (Figure 2B). Meanwhile the left primary visual cortex of the sham control group displayed no significant changes in theta medium-gamma PAC across the three stimulation stages (transient *P* = 0.131, post-stimulation 1 *P* = 0.056 and post-stimulation 2 *P* = 0.061) (Supplementary Figures 1A,B).

### Both Medium-Gamma and High-Gamma Oscillations Are Coupled to Theta Waves in the Prefrontal Cortex Following Retinal Electrical Stimulation

PAC characteristics of LFOs and HFOs have been demonstrated to play major roles in cognitive neuronal networks of the prefrontal cortex (Andino-Pavlovsky et al., 2017). This prompted us to analyze the changes in PAC of the left prefrontal cortex of rd10 mice in response to varying the stimulation current of TES. Similar to the left primary visual cortex, we monitored PAC features during different stages of TES (transient, post-stimulation 1 and post-stimulation 2) in all experimental groups (400, 500, and 600 μA and sham control), subsequently we compared the results with the corresponding baseline (pre-stimulation stage) PAC of rd10 mice. Unlike the left primary visual cortex for the transient stage, the left prefrontal cortex revealed significant increase for both theta medium-gamma (*P* = 0.012, *P* = 0.01, and *P* = 0.007) and high-gamma (*P* = 0.019, 0.013, and 0.007) PAC in 400, 500, and 600 μA stimulation groups, respectively, compared with the corresponding pre-stimulation PAC results (Figures 2C,D). Furthermore, for 1-week post-stimulation (post-stimulation stage 1), the left prefrontal cortex displayed significantly enhanced theta medium-gamma (*P* = 0.001, 0.002, and 0.005) and high-gamma (*P* = 0.01, 0.011, and 0.006) PAC, respectively, across all stimulation groups (400, 500, and 600 μA) compared with the corresponding PAC results at the pre-stimulation stage (Figures 2C,D).

We observed that for 2-weeks post-stimulation (post-stimulation stage 2), only rd10 mice stimulated with 400 μA maintained the significant increase in both theta medium-gamma (*P* = 0.01) and theta high-gamma PAC (*P* = 0.014) compared with the corresponding pre-stimulation PAC results (Figures 2C,D). In this regard rd10 mice stimulated with 500 and 600 μA, respectively, showed no significant change in theta-medium gamma (500 μA *P* = 0.486; 600 μA *P* = 0.213) and high-medium gamma (500 μA *P* = 0.455; 600 μA *P* = 0.526) PAC compared with the corresponding pre-stimulation PAC results (Figures 2C,D). Similar to the left primary visual cortex, in the left prefrontal cortex of rd10 mice stimulated with 400 μA, there was no significant change (Figure 2D) in theta medium-gamma (*P* = 0.172) and theta high-gamma (*P* = 0.683) PAC between post-stimulation stage 1 and post-stimulation stage 2. However, for both 500 and 600 μA groups, theta medium-gamma (500 μA *P* = 0.002; 600 μA *P* = 0.001) and theta high-gamma (500 μA *P* = 0.011; 600 μA *P* = 0.001) PAC in post-stimulation stage 2 was significantly reduced when compared to the corresponding PAC results in post-stimulation stage 1 (Figure 2D). Similar to the left primary visual cortex all stimulation stages, the left prefrontal cortex of the sham control group displayed no significant changes in theta medium-gamma (transient *P* = 0.197, post-stimulation 1 *P* = 0.035 and post-stimulation 2 *P* = 0.026) and theta high-gamma (transient *P* = 0.03, post-stimulation 1 *P* = 0.935 and post-stimulation 2 *P* = 0.985) PAC (Supplementary Figures 1C,D).

### Prolonged Transcorneal Electrical Stimulation Stimulates a Marked Increase of Coherence Between the Prefrontal Cortex and Primary Visual Cortex

**FIGURE 3:**
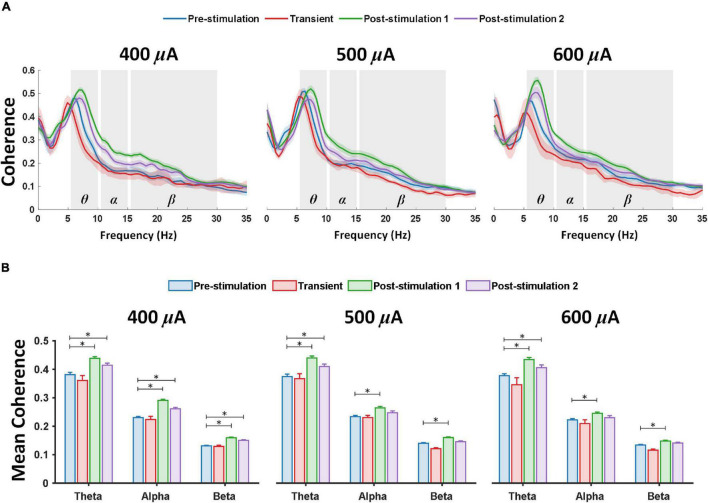
Theta, alpha and beta coherence is markedly elevated between the left prefrontal cortex and left primary visual cortex after retinal electrical stimulation. **(A)** Coherence spectrum showed prominent enhancement in theta, alpha and beta frequency bands; the shaded area depicts SEM. **(B)** Mean coherence values over two ECoG channels (left prefrontal cortex and primary visual cortex) from 400 μA stimulation (*n* = 6), 500 μA stimulation (*n* = 6), 600 μA stimulation (*n* = 6) during pre-stimulation (baseline), transient stimulation, post-stimulation stage 1 and post-stimulation stage 2. * Significant (*P* < 0.025); Error bar denotes SEM.

In addition to prominent increase in PAC, a large increase of coherence across theta, alpha and beta frequency band, respectively, was detected between the left prefrontal cortex and left primary visual cortex at various stimulation stages and current amplitudes of retinal stimulation (Figure 3A). For 1-week post-stimulation (post-stimulation stage 1), rd10 mice stimulated with 400, 500, and 600 μA all displayed significantly enhanced mean coherence in theta (*P* < 0.001, *P* < 0.001, and *P <* 0.001) alpha (*P* < 0.001, *P* = 0.001, and *P* = 0.017) and beta (*P* < 0.001, *P* < 0.001, and *P* = 0.001) oscillations between the left prefrontal cortex and left primary visual cortex (Figure 3B). This high coherence between the left prefrontal cortex and left primary visual cortex was maintained in theta (*P* = 0.014, *P* = 0.019, and *P <* 0.016) oscillations across all stimulation groups (400, 500, and 600 μA) for 2-weeks post-stimulation (post-stimulation stage 2) (Figure 3B). Furthermore, during the same period (post-stimulation stage 2) only rd10 mice stimulated with 400 μA significantly maintained the enhanced mean coherence between the left prefrontal cortex and left primary visual cortex in alpha (*P* < 0.001) and beta (*P* < 0.001) waves but this long-lasting enhancement was not seen in the alpha and beta waves of both 500 μA (*P* = 0.304, *P* = 0.494) and 600 μA (*P* = 0.768, *P* = 0.253) stimulation groups (Figure 3B).

It is worth noting that transient pTES did not elicit significant changes in theta, alpha and beta mean coherence between the left prefrontal cortex and left primary visual cortex across 400 μA (*P* = 0.510, *P* = 0.895, and *P* = 0.983), 500 μA (*P* = 0.974, *P* = 0.992, and *P* = 0.372), 600 μA (*P* = 0.266, *P* = 0.666, and *P* = 0.441) of rd10 mice (Figure 3B). Again, across the three stimulation stages (transient, post-stimulation stage 1 and post-stimulation stage 2) 400 μA stimulation showed no significant change in mean coherence of delta (*P* = 0.985, *P* = 0.378, and *P* = 0.835), low gamma (*P* = 0.961, *P* = 0.657, and *P* = 0.248), medium gamma (*P* = 0.349, *P* = 0.659, and *P* = 0.321), high gamma (*P* = 0.348, *P* = 0.144, and *P* = 0.166) and ultra-high gamma (*P* = 0.325, *P* = 0.224, and *P* = 0.543) compared with the corresponding pre-stimulation results (Supplementary Figure 2). Similarly, across the three stimulation stages (transient, post-stimulation stage 1 and post-stimulation stage 2) 500 μA stimulation showed no significant change in mean coherence of delta (*P* = 0.563, *P* = 0.957, and *P* = 0.995), low gamma (*P* = 0.403, *P* = 0.311, and *P* = 0.921), medium gamma (*P* = 0.827, *P* = 0.935, and *P* = 0.731), high gamma (*P* = 0.924, *P* = 0.716, and *P* = 0.692) and ultra-high gamma (*P* = 0.932, *P* = 0.618, and *P* = 0.602) compared with the corresponding pre-stimulation results (Supplementary Figure 2). 600 μA stimulation followed the same trend as 400 and 500 μA stimulations with no significant change in mean coherence of delta (*P* = 0.601, *P* = 0.538, *P* = 0.667), low gamma (*P* = 0.237, *P* = 0.285, and *P* = 0.156), medium gamma (*P* = 0.701, *P* = 0.084, and *P* = 0.061), high gamma (*P* = 0.322, *P* = 0.46, and *P* = 0.221) and ultra-high gamma (*P* = 0.467, *P* = 0.578, and *P* = 0.528) across the three stimulation stages (transient, post-stimulation stage 1 and post-stimulation stage 2), respectively, compared with the corresponding pre-stimulation results (Supplementary Figure 2). For sham control group, there was no significant change in mean coherence of delta (*P* = 0.984, *P* = 0.722, and *P* = 0.273), theta (*P* = 0.998, *P* = 0.991, and *P* = 0.147), alpha (*P* = 0.988, *P* = 0.986, and *P* = 0.0723) beta (*P* = 0.909, *P* = 0.681, and *P* = 0.317), low gamma (*P* = 0.861, *P* = 0.937, and *P* = 0.318), medium gamma (*P* = 0.981, *P* = 0.341, and *P* = 0.07), high gamma (*P* = 0.410, *P* = 0.068, and *P* = 0.093) and ultra-high gamma (*P* = 0.035, *P* = 0.08, and *P* = 0.049) oscillations across the three stimulation stages (transient, post-stimulation stage 1 and post-stimulation stage 2) (Supplementary Figure 3).

### Prefrontal Cortex-Primary Visual Cortex Connectivity of Theta, Alpha and Beta Waves After Prolonged Transcorneal Electrical Stimulation Exceed Baseline Feedforward and Feedback Directional Connectivity

**FIGURE 4:**
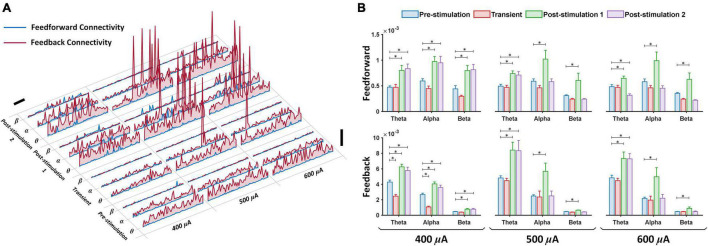
Corticocortical directional connectivity enhancement following retinal electrical stimulation. **(A)** Time course of feedforward (blue lines) and feedback (red lines) directional connectivity for theta, alpha and beta bands, respectively, during pre-stimulation (10 min), transient stimulation (10 min), post-stimulation stage 1 (10 min) and post-stimulation stage 2 (10 min). Feedforward (left primary visual cortex to left prefrontal cortex) and feedback (left prefrontal cortex to left primary visual cortex); vertical scale bar, 0.01, horizontal scale bar, 100 sec. **(B)** The average feedforward (upper panel) and feedback (lower panel) directional connectivity at three frequency bands from 400 μA stimulation (*n* = 6), 500 μA stimulation (*n* = 6), 600 μA stimulation (*n* = 6) was computed during pre-stimulation (baseline), transient stimulation, post-stimulation stage 1 and post-stimulation stage 2. Feedforward (left primary visual cortex to left prefrontal cortex) and feedback (left prefrontal cortex to left primary visual cortex). * Significant (*P* < 0.025); Error bar denotes SEM.

Presence of brain oscillations in the awake state has been associated with feedforward and feedback connectivity, portraying directional flow of neuronal information (Michalareas et al., 2016). Feedforward (left primary visual cortex to left prefrontal cortex) and feedback (left prefrontal cortex to left primary visual cortex) connectivity analyses were applied to the eight frequency bands under various stimulation current amplitudes (400, 500, and 600 μA) and different stages of stimulation (pre-stimulation, transient, post-stimulation stage 1 and post-stimulation stage 2) (Figures 4A,B). In the time series plot, we observed that all test experimental groups (400, 500, and 600 μA) displayed changes in feedforward (blue lines) and feedback (red lines) directional connectivity for theta, alpha and beta bands at different stages of stimulation (Figure 4A). For the feedforward direction, transient pTES largely showed no significant change in theta, alpha and beta oscillations of 400 μA (*P* = 0.771, *P* = 0.628, and *P* = 0.563), 500 μA (*P* = 0.987, *P* = 0.781, and *P* = 0.905), and 600 μA (*P* = 0.994, *P* = 0.809, and *P* = 0.592) stimulations, respectively, compared with the corresponding pre-stimulation directional connectivity (Figure 4B upper panel). This same trend was observed in the respective theta, alpha and beta feedback directional connectivity of rd10 mice stimulated with 500 μA (*P* = 0.992, *P* = 0.939, and *P* = 0.362), and 600 μA (*P* = 0.974, *P* = 0.996, and *P* = 0.916) compared to the corresponding pre-stimulation directional connectivity (Figure 4B lower panel). For 400 μA stimulation group, transient pTES yielded no significant change in beta (*P* = 0.909) feedback directional connectivity while simultaneously causing significant decreased theta (*P* < 0.001) and alpha (*P* < 0.001) feedback directional connectivity, respectively, compared with the corresponding pre-stimulation directional connectivity (Figure 4B lower panel).

At 1-week post-stimulation (post-stimulation stage 1), we observed striking significant increase in feedforward direction connectivity of theta, alpha and beta oscillations for 400 μA (*P* = 0.009, *P* = 0.013, *P* = 0.008), 500 μA (*P* = 0.004, *P* = 0.01, *P* = 0.017), and 600 μA (*P* = 0.014, *P* = 0.012, *P* = 0.015) stimulations, respectively, compared with the corresponding pre-stimulation directional connectivity (Figure 4B upper panel). Similar significant increase was also observed in the feedback direction of theta, alpha and beta oscillations for 400 μA (*P* < 0.001, *P* < 0.001, *P* < 0.001), 500 μA (*P* = 0.017, *P* = 0.013, *P* < 0.001), and 600 μA (*P* = 0.015, *P* = 0.021, *P* = 0.02) stimulations, respectively, compared with the corresponding pre-stimulation directional connectivity (Figure 4B lower panel). This analysis also uncovered that at 2-weeks after the start of the 7-days pTES (post-stimulation stage 2), rd10 mice stimulated with 400 μA maintained significant increase in feedforward connectivity of theta (*P* = 0.003), alpha (*P* = 0.022) and beta (*P* = 0.005) oscillations, respectively, and also feedback connectivity of theta (*P* = 0.006), alpha (*P* = 0.02) and beta (*P* = 0.021) oscillations, respectively, compared with the corresponding pre-stimulation connectivity (Figure 4B upper and lower panels). In comparison to the pre-stimulation stage, we additionally observed that at the post-stimulation stage 2, both 500 and 600 μA stimulation groups significantly maintained the elevated increase in both feedforward (for 500 μA stimulation *P* = 0.014, for 600 μA stimulation *P* = 0.01) and feedback theta connectivity (for 500 μA stimulation *P* = 0.021, for 600 μA stimulation *P* = 0.017). However, this feature was lost in the feedforward direction of both alpha (for 500 μA stimulation *P* = 0.9, for 600 μA stimulation *P* = 0.782) and beta (for 500 μA stimulation *P* = 0.953, for 600 μA stimulation *P* = 0.445) and the feedback directions of both alpha (for 500 μA stimulation *P* = 0.91, for 600 μA stimulation *P* = 0.9) and beta (for 500 μA stimulation *P* = 0.748, for 600 μA stimulation *P* = 0.95) (Figure 4B upper and lower panels).

For analysis of the other brain oscillations, across the three stimulation stages (transient, post-stimulation stage 1 and post-stimulation stage 2) 400 μA stimulation showed no significant change in feedforward connectivity of delta (*P* = 0.996, *P* = 0.768 and *P* = 0.985), low gamma (*P* = 0.738, *P* = 0.213, and *P* = 0.232), medium gamma (*P* = 0.91, *P* = 0.95, and *P* = 0.982), high gamma (*P* = 0.763, *P* = 0.8012, and *P* = 0.708) and ultra-high gamma (*P* = 0.6, *P* = 0.077, and *P* = 0.116) compared with the corresponding pre-stimulation results (Supplementary Figure 4 upper panel). In the feedback direction there was also no significant change in the connectivity of delta (*P* = 0.904, *P* = 0.961, and *P* = 0.973), low gamma (*P* = 0.26, *P* = 0.433, and *P* = 0.301), medium gamma (*P* = 0.703, *P* = 0.751, and *P* = 0.71), high gamma (*P* = 0.234, *P* = 0.218, and *P* = 0.281) and ultra-high gamma (*P* = 0.071, *P* = 0.045, and *P* = 0.031) compared with the corresponding pre-stimulation results (Supplementary Figure 4 lower panel). For 500 μA stimulation feedforward connectivity also displayed no significant change across the three stimulation stages (transient, post-stimulation stage 1 and post-stimulation stage 2) of delta (*P* = 0.607, *P* = 0.917, and *P* = 0.919), low gamma (*P* = 0.948, *P* = 0.395, and *P* = 0.803), medium gamma (*P* = 0.741, *P* = 0.052, and *P* = 0.071), high gamma (*P* = 0.982, *P* = 0.972, and *P* = 0.98) and ultra-high gamma (*P* = 0.687, *P* = 0.736, and *P* = 0.497) compared with the corresponding pre-stimulation results (Supplementary Figure 4 upper panel). In this regard, feedback connectivity for 500 μA stimulation also achieved no significant change in delta (*P* = 0.949, *P* = 0.968, and *P* = 0.980), low gamma (*P* = 0.957, *P* = 0.98, and *P* = 0.981), medium gamma (*P* = 0.969, *P* = 0.967, and *P* = 0.933), high gamma (*P* = 0.961, *P* = 0.875, and *P* = 0.952) and ultra-high gamma (*P* = 0.826, *P* = 0.372, and *P* = 0.464) oscillations compared with the corresponding pre-stimulation results (Supplementary Figure 4 lower panel). 600 μA stimulation followed the same trend as 400 and 500 μA stimulations with no significant change in feedforward connectivity and feedback connectivity compared with the corresponding pre-stimulation results (Supplementary Figure 4 upper and lower panels). The following *P-*values were obtained across the three stimulation stages for 600 μA stimulation in the feedforward direction; Delta (*P* = 0.994, *P* = 0.833, and *P* = 0.908), low gamma (*P* = 0.947, *P* = 0.968, and *P* = 0.952), medium gamma (*P* = 0.486, *P* = 0.707, *P* = 0.264), high gamma (*P* = 0.702, *P* = 0.615, *P* = 0.185) and ultra-high gamma (*P* = 0.967, *P* = 0.366, and *P* = 0.346). Meanwhile in the feedback direction the *P-*values across the three stimulation stages for 600 μA stimulation were Delta (*P* = 0.981, *P* = 0.904, and *P* = 0.971), low gamma (*P* = 0.913, *P* = 0.9, and *P* = 0.983), medium gamma (*P* = 0.977, *P* = 0.948, *P* = 0.906), high gamma (*P* = 0.904, *P* = 0.878, *P* = 0.969) and ultra-high gamma (*P* = 0.929, *P* = 0.766, *P* = 0.766).

Meanwhile rd10 mice that received no pTES (sham control group) showed no significant difference in feedforward connectivity across all assessed oscillations and three stages of stimulation compared to the corresponding pre-stimulation results (Supplementary Figures 5, 6) (Delta *P* = 0.979, *P* = 0.931, and *P* = 0.972; Theta *P* = 0.971, *P* = 0.833 and *P* = 0.958; Alpha *P* = 0.903, *P* = 0.511, and *P* = 0.283; Beta *P* = 0.947, *P* = 0.843, and *P* = 0.968; Low gamma *P* = 0.815, *P* = 0.754, and *P* = 0.341; Medium gamma *P* = 0.477, *P* = 0.195, and *P* = 0.26; High gamma *P* = 0.382, *P* = 0.199, and *P* = 0.374; Ultra-high gamma *P* = 0.828, *P* = 0.858, and *P* = 0.967). Similarly, this feature was also observed in the feedback direction of the sham control group across all assessed oscillations and across the three stages of stimulation compared to the corresponding pre-stimulation results (Delta *P* = 0.971, *P* = 0.983, and *P* = 0.961; Theta *P* = 0.409, *P* = 0.98, and *P* = 0.901; Alpha *P* = 0.449, *P* = 0.912, and *P* = 0.904; Beta *P* = 0.953, *P* = 0.9234, and *P* = 0.9524; Low gamma *P* = 0.663, *P* = 0.695, and *P* = 0.352; Medium gamma *P* = 0.921, *P* = 0.464, and *P* = 0.724; High gamma *P* = 0.781, *P* = 0.876, and *P* = 0.984; Ultra-high gamma *P* = 0.342, *P* = 0.987, and *P* = 0.417).

## Discussion

### Electrocorticography Recording in Awake Mice and Circadian Impacts

Previous ECoG studies have reported that modulations in time of the day affect cognitive performance as well as neurophysiological activities of the brain (Blatter and Cajochen, 2007; García et al., 2012; Chellappa et al., 2018; Facer-Childs et al., 2018; Fisk et al., 2018). In other words, circadian rhythm plays a key role in biological functioning in living systems. Intrinsic factors such as sleep-wake cycle and external factors such as lighting conditions also play unique roles in controlling circadian rhythm of biological systems (Waterhouse et al., 2012; Hickie et al., 2013; Musiek et al., 2015; Blume et al., 2019; Bano-Otalora et al., 2021). In the design of our study, we considered these factors and ensured that appropriate precautions were taken to avoid disruption of circadian rhythms in all experimental animals. To achieve this, the experimental animals were exposed to 12 h light and 12 h dark phases. ECoG recording sessions were always performed in all rd10 mice at the same time period during the light phase and under ambient lighting conditions. Care was also taken to ensure that a healthy body weight of all mice was maintained. No sudden weight loss was observed in all animals during the study period.

It is also important to note that brain oscillations recorded from EEG/ECoG have been well recognized as a basis for cognitive capacity, memory, and learning in animals and humans (Pietto et al., 2018; Buenrostro-Jáuregui et al., 2020; Šneidere et al., 2020). Therefore, the simultaneous monitoring of behavioral changes and ECoG is particularly interesting in correlating data between brain activity and task-related behaviors (Miller, 2019). Apart from assuring proper sleep-wake cycle, maintenance of body weight and proper lighting conditions in experimental animals during our study; the behavior of the animals was carefully monitored during ECoG recording sessions. With regards to the animal behavior, all rd10 animals expressed active behaviors including manifesting free movement, coordinated exploration of their surrounding environment, reaching for food/water, paw licking and other grooming behaviors. No animal showed signs of distress or physical signs of depressive behavior during ECoG recording. All the aforementioned manifestations satisfied our criteria for an active awake rodent.

### Prolonged Transcorneal Electrical Stimulation-Triggered Enhancement in Functional and Directional Connectivity Is Maintained in Visual and Non-visual Brain Regions

The data reported in the present study demonstrate that pTES triggers a long-lasting enhancement of coordinated theta, alpha and beta waves, which displays high levels of interregional coherence and connectivity as well as synchronized phase-amplitude coupling characteristics between theta and gamma oscillations in rd mice. This long-lasting enhancement in PAC, coherence and directional connectivity was observed not only in the visual region (primary visual cortex) of stimulated animals, but also featured in the non-visual region (prefrontal cortex). Invariably, this suggests that electrical stimulation of the retina does not only affect neurons of the primary visual cortex but also activates connectivity networks in the prefrontal cortex of rd animals.

In both primates and rodents, the prefrontal cortex has been reported to be involved in cognitive processes including working memory, attention, decision-making and cognitive flexibility. Arising from neuroanatomy, the prefrontal cortex has circuits that integrate diverse neural information from other cortical and subcortical regions including the basal ganglia, hippocampus and amygdala. The aforementioned design enables the prefrontal cortex exert highly coordinated cognitive functions that govern behavioral control. Working memory is defined as a system that enables short-term retainment (seconds to minutes time scale) of neural information for use in complex task completion (Funahashi, 2017; Jobson et al., 2021). The mPFC has been severally implicated in the working memory process. For example, early studies in rodents have demonstrated that mPFC lesions result in delayed response in task related behaviors and clear deficits for visual objects information (Kesner et al., 1996; Kahn et al., 2012). Attention is a complex cognitive process that allows the brain to effectively assign sensory resources for instantaneous goals while disregarding other irrelevant sensory inputs. The crucial function of the prefrontal cortex has also been demonstrated in rodent-based attention tasks which resulted in choice accuracy reduction and slow responses for rats with lesioned mPFC (Chudasama et al., 2003). Decision-making is the executive capacity of an animal to choose an advantageous response from several possible choices. Evidence from neurophysiological recordings have proven that the orbitofrontal cortex (OFC) and the mPFC (specifically the anterior cingulate cortex) work together to update, evaluate and translate choice options into final decision actions in non-human primates (Hayden and Platt, 2010; Sul et al., 2010). Based on the aforementioned evidence of the crucial roles of the prefrontal cortex in cognition, we suggest that in our present study each of the properties of theta, alpha and beta oscillations observed in both prefrontal cortex and primary visual cortex indicate highly coordinated neural activities in the brain following retinal electrical stimulation. Indeed theta coherence and connectivity has been reported to be important in cognitive performance of task-related functions between the frontal regions, occipital, parietal, motor areas and sub-thalamic nucleus of the brain (Nurislamova et al., 2019). Alpha oscillations have been implicated in the directional coordination of control processes including task-related information. Specifically, these alpha waves have been severally associated with inhibitory functions where they have been shown to play crucial roles in reducing irrelevant neural connections thus enabling the brain to focus more on progressive connections (Wolff et al., 2017). Pre-clinical studies in retinal degeneration rodents have reported irrelevant spontaneous firing as neurophysiological-markers of the retinal degeneration pathology (Wang et al., 2016). Our present study showed an increased surge of alpha coherence and alpha directional connectivity between the prefrontal cortex and visual cortex which suppresses the interfering spontaneous firing in rd neurons and possibly establishes a more coordinated communication between neurons in the prefrontal cortex and visual cortex. Beta oscillations have previously been tagged to index changes in underlying neural activities in the sensorimotor cortex (Schmidt et al., 2019). In the present study, the increased surge of beta oscillations in coherence and connectivity measures may possibly be involved in global integration of newly established neural network connections that occurred in the rd brain during post-stimulation stage 1. Sham control group that did not received pTES displayed no change in the analyzed indices of brain coherence and connectivity which confirms that pTES is responsible for the observed alterations in brain coherence and connectivity of the stimulated test groups.

Based on the duration of the present study, the observed effects of pTES in both primary visual cortex and prefrontal cortex do not occur only 1-week after the start of pTES but appear to have maintained impacts for up to 2-weeks after the start of the 7-days pTES. This suggests that given the time period of the present study, pTES could possibly lead to the formation and strengthening of new pathways of synchronous neural connections in rd brain after electrical stimulation. Although all stimulation currents enhanced and maintained theta-gamma modulation in one or more stimulation stages, 400 μA showed the greatest maintenance effect followed by 500 μA and lastly 600 μA compared to the corresponding pre-stimulation PAC results. This phenomenon could possibly be explained by the higher tendency toward a saturated evoked response in the primary visual cortex neurons on application of 500 and 600 μA stimulation current amplitudes, respectively, compared with 400 μA (Supplementary Figure 7). Subsequently, due to coherence and connectivity between the primary visual cortex and prefrontal cortex, the high tendency toward a saturated evoked response in the primary visual cortex neurons with 500 and 600 μA current stimulation extended to the prefrontal cortex and induced lower enhancement of brain coherence and connectivity on application of 500 and 600 μA stimulations compared with 400 μA (as already described in the “Result” section of the present study). Our data therefore indicates that the applied pTES facilitated specific cortical systems by progressive interactions with neuronal oscillators that are functionally coupled based on the applied current amplitude. As PAC has been reported to be critical in transfer of neural information across a wide range of spatio-temporal scales and integrates global communication among neural populations, it is speculated to have high relevance for cognitive processing (Lara et al., 2018; Munia and Aviyente, 2019). More compelling evidence recently supports the above report by highlighting the functional relevance of phase-amplitude coupling in visual perception (Zazio et al., 2020), visuomotor mapping (Tzvi et al., 2016), learning and memory consolidation (Cox et al., 2020).

### Potential Involvement of the Pulvinar Nucleus in Prolonged Transcorneal Electrical Stimulation-Induced Enhancement of Functional and Directional Connectivity

Several studies that investigate the cellular basis of visual perception often consider direct corticocortical circuits as the predominant pathway for neuronal connectivity. Aside this corticocentric view of neural information transmission in the CNS, there is now emerging evidence for the crucial roles of indirect pathways that involve the pulvinar nucleus (Halassa and Kastner, 2017). In mammals, the pulvinar is the largest extrageniculate nucleus of the thalamus that provides an additional communication route between the visual regions of the cortex. Briefly, the pulvinar is bi-synaptically linked to the primary visual cortex and extrastriate visual cortical regions *via* cortico-thalamo-cortical projections (Arcaro et al., 2015; Bennett et al., 2019). This design strategically positions the pulvinar at the middle of several pathways between the thalamus and the cortex. Consequently, the pulvinar has been reported to play important roles in the contextual and multi-sensory processing (Ibrahim et al., 2016; Roth et al., 2016; Chou et al., 2020; Fang et al., 2020) and synchronization among cortical regions (Saalmann et al., 2012; Schmid et al., 2012). Indeed, the pulvinar has previously been implicated in the control of neuronal oscillations particularly in the visual cortex. Recall that synchronization of brain oscillations is a key marker for cortical processing. Accordingly, electrophysiological recordings from anesthetized cats have demonstrated that inactivation of the pulvinar induces desynchronization in areas 17 or 18 of the visual cortex (Wróbel et al., 2007; Cortes et al., 2020). The pulvinar contribution to corticocortical communication between higher visual areas (V4 and above) has also been investigated in recent studies (Saalmann et al., 2012; Fiebelkorn et al., 2019). Results from these studies reveal the occurrence of alpha-driven pulvinar synchronized activity between interconnected cortical regions under attention-related tasks. In addition, the pulvinar has been reported to consist of two major cell types; the excitatory “relay” neurons which project to the cerebral cortex and the intrinsic inhibitory interneurons which are localized within the nucleus. Taking into consideration the aforementioned functional and anatomical characteristics of the pulvinar, we hypothesize that pTES produces the beneficial enhancement of brain connectivity by possible indirect activation of the pulvinar to establish a physiological balance in cortical excitation and inhibition. However, this hypothesis warrants further investigation for confirmation.

### Implications for Retinitis Pigmentosa

In mammals, emotions have been described as canonical responses to situations that are linked to survival or well-being of the animal concerned. The neural circuits involved in emotional representation seems to functionally coordinate both visual and non-visual brain regions (Wyczesany et al., 2015; Kragel et al., 2019). For example, depression which has been found to originate from abnormal functioning of the medial prefrontal cortex has also been observed in visually impaired patients (Davey et al., 2017; Zheng et al., 2017). Specifically, retinitis pigmentosa has been reported to be emotionally devastating in affected individuals due to the anticipation of vision loss and the eventual blindness causes profound distress (Hahm et al., 2008). These factors alone increase retinitis pigmentosa patients’ vulnerability to depression. In fact, a higher prevalence of anxiety and depression has been reliably documented in retinitis pigmentosa patients compared with the general population without vision loss (Moschos et al., 2015). Consequently, clinical interventions that provide efficient therapies for depression treatment have become of core necessity in order to improve the overall quality of life in retinitis pigmentosa patients. In this regard, pTES has been found to attenuate depressive symptoms from rodents with visual deficit (Yu et al., 2019, 2021). This finding in addition to our present results is suggestive that pTES holds strong possibility of modulating higher cortical functions including pathways of cognition, emotion, awareness, and memory in retinitis pigmentosa patients. However, further experimental studies to confirm this possibility remains to be investigated.

In addition to the beneficial effect of pTES in depression, we believe that pTES can also be used to enhance visual-related behaviors specifically visual attention. Previous studies on visual detection tasks in healthy humans have applied tDCs to modulate spatial attention by enhancing the ratio of neural activity between the left and right hemispheres of the brain (Ni et al., 2019; Andres et al., 2020). In another study tDCs applied in the alpha oscillatory spectrum was reported to facilitate cognitive control and improve performance in visual attention tasks (Clayton et al., 2019). Similar to the above studies, our present work shows clear evidence of pTES-induced enhancement in alpha oscillations and as previously stated, alpha oscillations have been shown to help the brain focus on relevant neuronal signals. Thus, like tDCs our present study suggests that pTES could possibly improve visual attention and thus may be applied for functional rehabilitation of visual negligence. However, the aforementioned claims can only be ascertained by more detailed experimentation.

Collectively, the data of the present study shows that pTES drastically improves network coherence and connectivity patterns both at the level of cross-oscillatory interaction and at the level of functional and directional inter-regional communication between the primary visual cortex and prefrontal cortex. To the best of our knowledge, this makes our study the first of its kind to demonstrate that pTES is able to alter coherence and connectivity measures of non-visual areas, in this case the prefrontal cortex. On this basis, a fundamental aspect of our study is that it provides an insight for which researchers in the field could apply pTES as a novel neuromodulatory approach to target both visual and non-visual regions of the brain directly from the mammalian eye. Thus, this serves as a simplistic strategy that is capable of opening new horizons for unlocking previously unexplored brain pathways with possible beneficial outcomes.

## Data Availability Statement

The original contributions presented in the study are included in the article/Supplementary Material, further inquiries can be directed to the corresponding author/s.

## Ethics Statement

The animal study was reviewed and approved by the Animal Research Ethics Sub-Committee at City University of Hong Kong.

## Author Contributions

SA and LLHC designed the study and wrote the manuscript. SA performed the *in vivo* experiments. SA and AE analyzed the data. All authors contributed to the article and approved the submitted version.

## Author Disclaimer

Any opinions, findings, conclusions in this publication do not reflect the views of the Government of the Hong Kong Special Administrative Region, the Innovation and Technology Commission or the Innovation and Technology Fund Research Projects Assessment Panel.

## Conflict of Interest

The authors declare that the research was conducted in the absence of any commercial or financial relationships that could be construed as a potential conflict of interest.

## Publisher’s Note

All claims expressed in this article are solely those of the authors and do not necessarily represent those of their affiliated organizations, or those of the publisher, the editors and the reviewers. Any product that may be evaluated in this article, or claim that may be made by its manufacturer, is not guaranteed or endorsed by the publisher.

## Supplementary Material

Supplementary Figure 1 **(A)** Phase-amplitude comodulograms computed for ECoG signals obtained from the left primary visual cortex of sham control rd10 mice. **(B)** Mean *MI* values for phases of slow theta waves (5.5–10 Hz) and amplitudes of fast medium-gamma oscillations (60–115 Hz) in the left primary visual cortex. **(C)** Phase-amplitude comodulograms computed for ECoG signals obtained from the left prefrontal cortex of sham control rd10 mice. **(D)** Mean *MI* values for phases of slow theta waves (5.5–10 Hz) and amplitudes of both medium-gamma oscillations (60–115 Hz) and high-gamma oscillations (125–175 Hz) in the left prefrontal cortex. There was no significant change (*p* > 0.025) in theta medium-gamma phase amplitude coupling in the in the left primary visual cortex **(A,B)** of sham control rd10 mice (*n* = 6) across all four stimulation stages. Similarly, in the left prefrontal cortex **(C,D)** there was no significant change in phase amplitude coupling between theta medium-gamma (*p* > 0.025) and theta high-gamma bands (*p* > 0.025), respectively, across all four stimulation stages. *θ—γm*: Theta medium-gamma coupling; *θ—γh*: Theta high-gamma coupling. NS, not significant; Error bar denotes SEM.

Supplementary Figure 2 Delta and gamma coherence is unchanged between the left prefrontal cortex and left primary visual cortex after retinal electrical stimulation. Mean coherence values over two ECoG channels (left prefrontal cortex and primary visual cortex) from 400 μA stimulation (*n* = 6), 500 μA stimulation (*n* = 6), 600 μA stimulation (*n* = 6) during pre-stimulation (baseline), transient stimulation, post-stimulation stage 1 and post-stimulation stage 2. *γl* low-gamma; *γm* medium-gamma; *γh* high-gamma; *γu* ultra-gamma, NS, not significant (*P* > 0.025); Error bar denotes SEM.

Supplementary Figure 3 Mean coherence between the left prefrontal cortex and left primary visual cortex is unchanged (*p* > 0.025) across all analyzed oscillatory bands in sham control rd10 mice. Mean coherence values over two ECoG channels (left prefrontal cortex and primary visual cortex) from sham control rd10 mice (*n* = 6) during pre-stimulation (baseline), transient stimulation, post-stimulation stage 1 and post-stimulation stage 2. θ Theta; α alpha; β beta; *γl* low-gamma; *γm* medium-gamma; *γh* high-gamma; *γu* ultra-gamma, NS, not significant (*P* > 0.025); Error bar denotes SEM.

Supplementary Figure 4 Delta and gamma corticocortical directional connectivity remains unchanged after retinal electrical stimulation. The average feedforward (upper) and feedback (lower) directional connectivity at three frequency bands from 400 μA stimulation (*n* = 6), 500 μA stimulation (*n* = 6), 600 μA stimulation (*n* = 6) was computed during pre-stimulation (baseline), transient stimulation, post-stimulation stage 1 and post-stimulation stage 2. *γl* low-gamma; *γm* medium-gamma; *γh* high-gamma; *γu* ultra-gamma. Feedforward (left primary visual cortex to left prefrontal cortex) and feedback (left prefrontal cortex to left primary visual cortex). NS, not significant (*P* > 0.025); Error bar denotes SEM.

Supplementary Figure 5 Time course of feedforward (blue lines) and feedback (red lines) directional connectivity for theta, alpha and beta bands, respectively, from sham control rd10 mice (*n* = 6) during pre-stimulation (10 min), transient stage (10 min), post-stimulation stage 1 (10 min) and post-stimulation stage 2 (10 min). Feedforward (left primary visual cortex to left prefrontal cortex) and feedback (left prefrontal cortex to left primary visual cortex).

Supplementary Figure 6 Corticocortical directional connectivity across all analyzed bands remains unchanged in sham control rd10 mice (*n* = 6). The average feedforward (upper) and feedback (lower) directional connectivity across all analyzed frequency bands from the sham control group was computed during pre-stimulation (baseline), transient stimulation, post-stimulation stage 1 and post-stimulation stage 2. θ Theta; α alpha; β beta; *γl* low-gamma; *γm* medium-gamma; *γh* high-gamma; *γu* ultra-gamma; Feedforward (left primary visual cortex to left prefrontal cortex) and feedback (left prefrontal cortex to left primary visual cortex). NS, not significant; Error bar denotes SEM.

Supplementary Figure 7 Average dose response plot from the left primary visual cortex. The average response amplitudes were obtained from six rd10 mice.

Electrical evoked response across 19 current amplitudes and 10 Hz stimulation frequency, was fitted into sigmoid curves. The response amplitude was expressed as a percentage of the maximum EEP response. Effective concentration (EC) represents the effective response amplitude. Current amplitudes of 400, 500, and 600 μA elicited an effective dose response amplitude of 50, 70, and 85%, respectively, across all applied stimulation frequencies. Beyond 500 and 600 μA current stimulations, response amplitude only increases marginally as the curve approaches the saturation point.
