# Supplementary figures and images for: Transcorneal Electrical Stimulation Induces Long-Lasting Enhancement of Brain Functional and Directional Connectivity in Retinal Degeneration Mice
Source: Front Cell Neurosci. 2022 Feb 7;16:785199. doi: 10.3389/fncel.2022.785199 (PMC8860236; doi:10.3389/fncel.2022.785199)

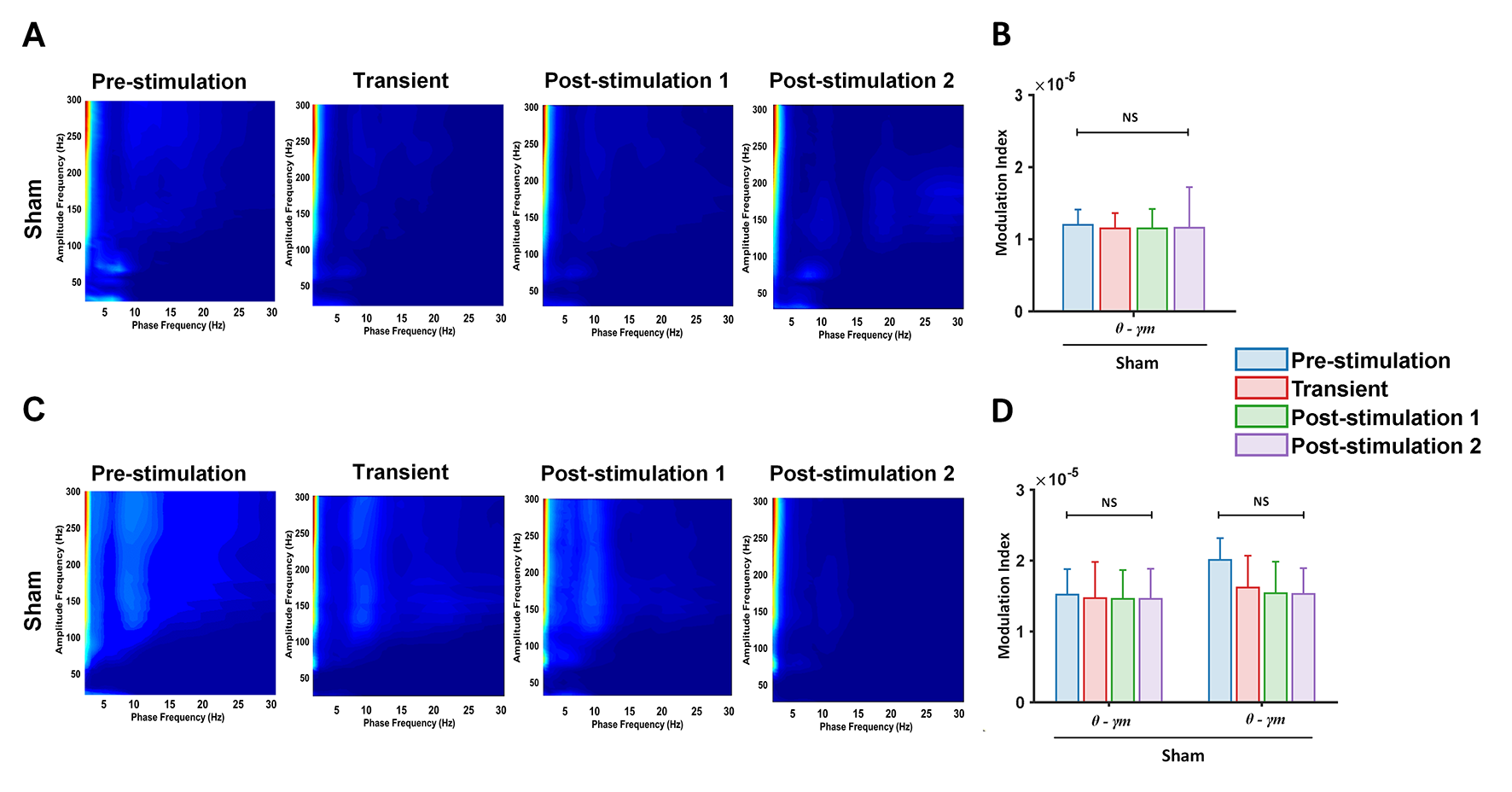

Supplement: Supplementary Figure 1 — (A) Phase-amplitude comodulograms computed for ECoG signals obtained from the left primary visual cortex of sham control rd10 mice. (B) Mean MI values for phases of slow theta waves (5.5–10 Hz) and amplitudes of fast medium-gamma oscillations (60–115 Hz) in the left primary visual cortex. (C) Phase-amplitude comodulograms computed for ECoG signals obtained from the left prefrontal cortex of sham control rd10 mice. (D) Mean MI values for phases of slow theta waves (5.5–10 Hz) and amplitudes of both medium-gamma oscillations (60–115 Hz) and high-gamma oscillations (125–175 Hz) in the left prefrontal cortex. There was no significant change (p > 0.025) in theta medium-gamma phase amplitude coupling in the in the left primary visual cortex (A,B) of sham control rd10 mice (n = 6) across all four stimulation stages. Similarly, in the left prefrontal cortex (C,D) there was no significant change in phase amplitude coupling between theta medium-gamma (p > 0.025) and theta high-gamma bands (p > 0.025), respectively, across all four stimulation stages. θ—γm: Theta medium-gamma coupling; θ—γh: Theta high-gamma coupling. NS, not significant; Error bar denotes SEM. [file Image_1.TIF]

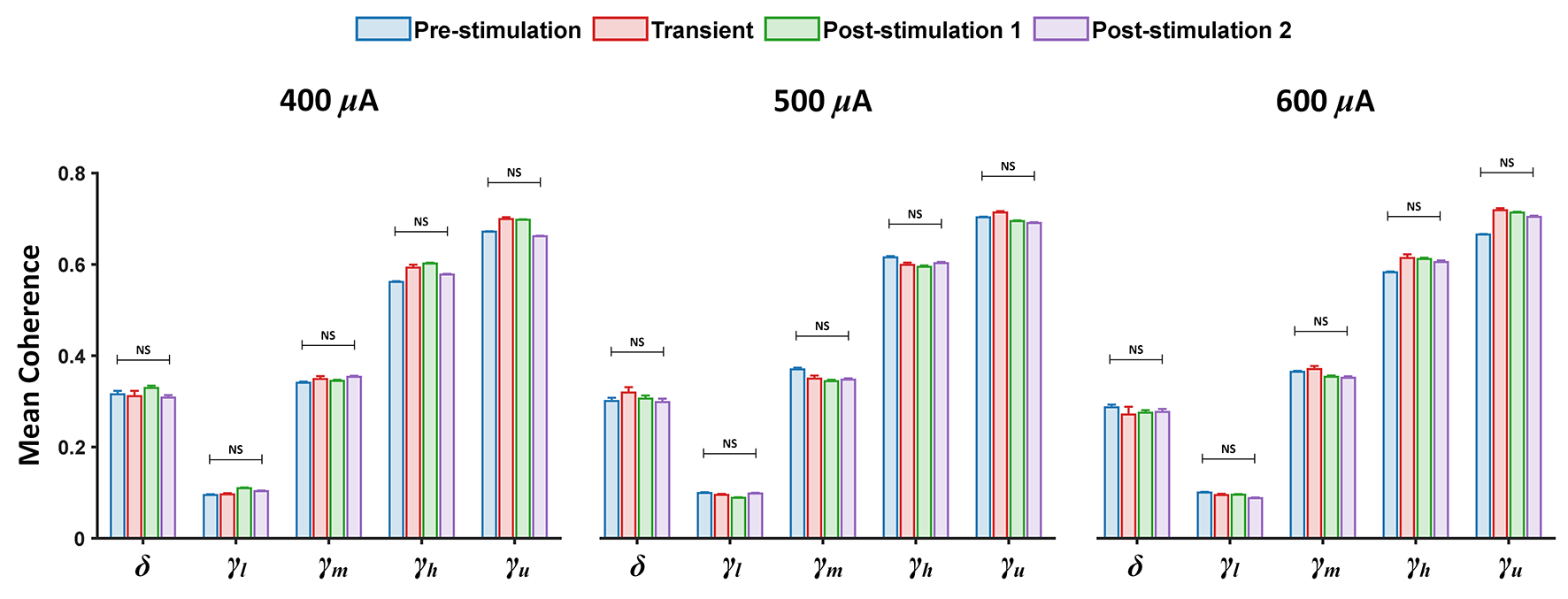

Supplement: Supplementary Figure 2 — Delta and gamma coherence is unchanged between the left prefrontal cortex and left primary visual cortex after retinal electrical stimulation. Mean coherence values over two ECoG channels (left prefrontal cortex and primary visual cortex) from 400 μA stimulation (n = 6), 500 μA stimulation (n = 6), 600 μA stimulation (n = 6) during pre-stimulation (baseline), transient stimulation, post-stimulation stage 1 and post-stimulation stage 2. γl low-gamma; γm medium-gamma; γh high-gamma; γu ultra-gamma, NS, not significant (P > 0.025); Error bar denotes SEM. [file Image_2.TIF]

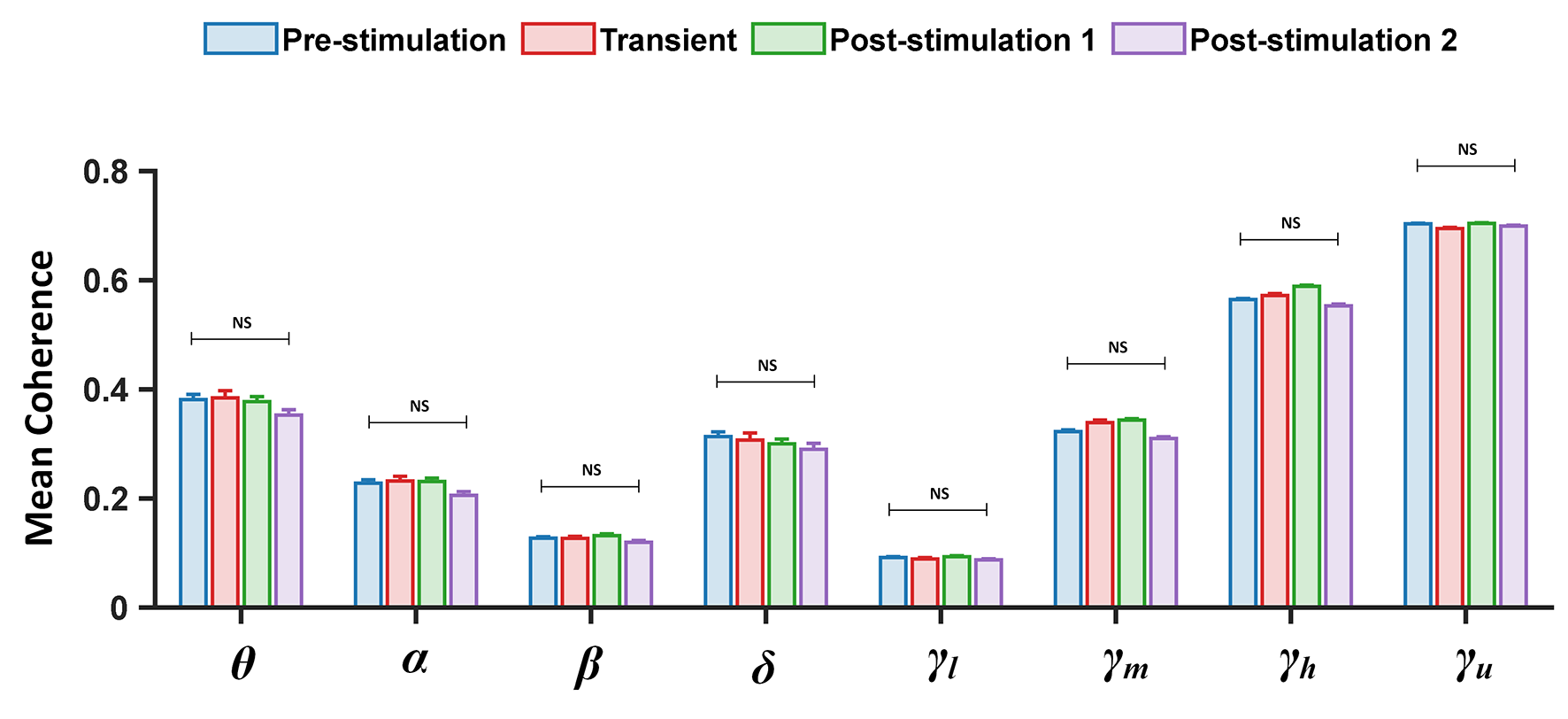

Supplement: Supplementary Figure 3 — Mean coherence between the left prefrontal cortex and left primary visual cortex is unchanged (p > 0.025) across all analyzed oscillatory bands in sham control rd10 mice. Mean coherence values over two ECoG channels (left prefrontal cortex and primary visual cortex) from sham control rd10 mice (n = 6) during pre-stimulation (baseline), transient stimulation, post-stimulation stage 1 and post-stimulation stage 2. θ Theta; α alpha; β beta; γl low-gamma; γm medium-gamma; γh high-gamma; γu ultra-gamma, NS, not significant (P > 0.025); Error bar denotes SEM. [file Image_3.TIF]

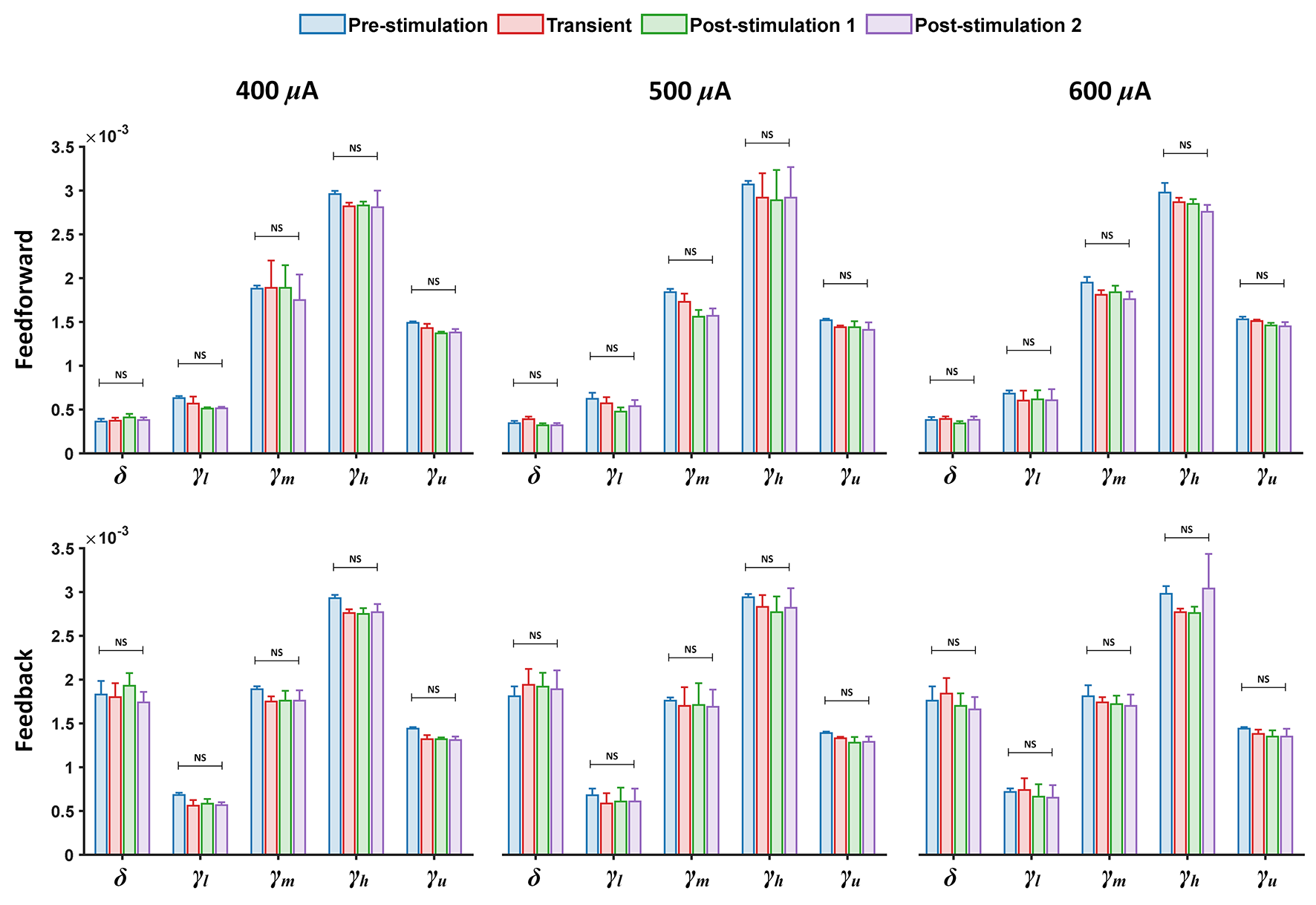

Supplement: Supplementary Figure 4 — Delta and gamma corticocortical directional connectivity remains unchanged after retinal electrical stimulation. The average feedforward (upper) and feedback (lower) directional connectivity at three frequency bands from 400 μA stimulation (n = 6), 500 μA stimulation (n = 6), 600 μA stimulation (n = 6) was computed during pre-stimulation (baseline), transient stimulation, post-stimulation stage 1 and post-stimulation stage 2. γl low-gamma; γm medium-gamma; γh high-gamma; γu ultra-gamma. Feedforward (left primary visual cortex to left prefrontal cortex) and feedback (left prefrontal cortex to left primary visual cortex). NS, not significant (P > 0.025); Error bar denotes SEM. [file Image_4.TIF]

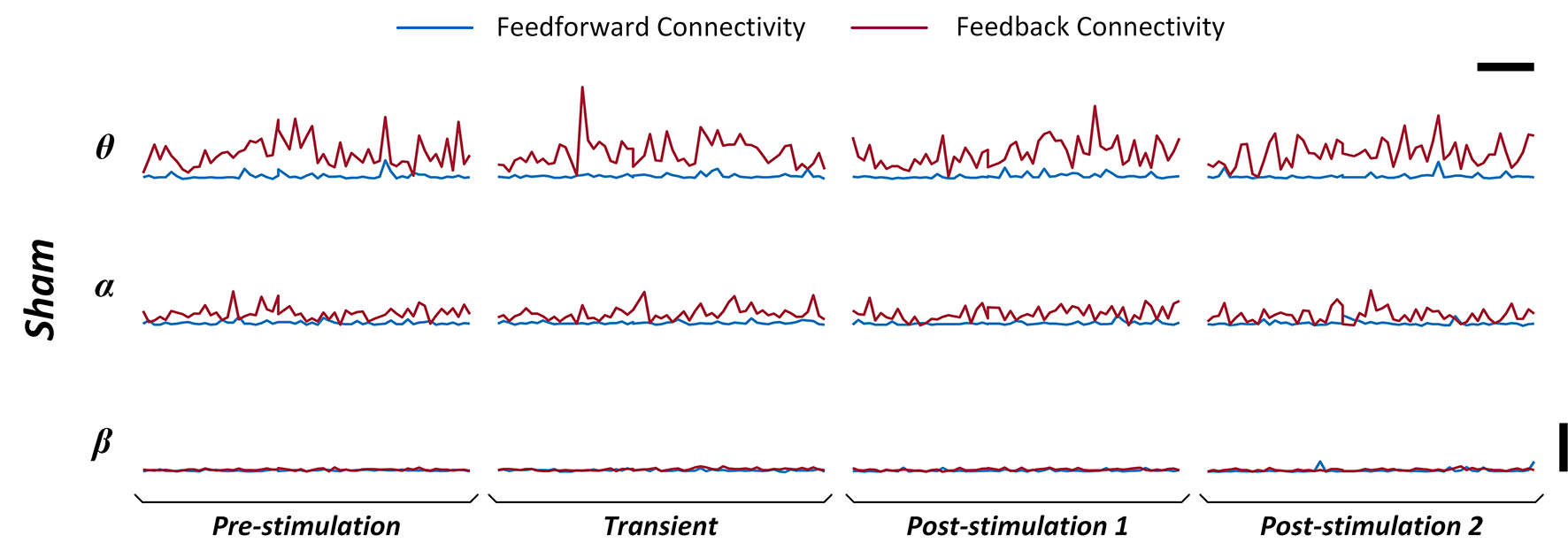

Supplement: Supplementary Figure 5 — Time course of feedforward (blue lines) and feedback (red lines) directional connectivity for theta, alpha and beta bands, respectively, from sham control rd10 mice (n = 6) during pre-stimulation (10 min), transient stage (10 min), post-stimulation stage 1 (10 min) and post-stimulation stage 2 (10 min). Feedforward (left primary visual cortex to left prefrontal cortex) and feedback (left prefrontal cortex to left primary visual cortex). [file Image_5.TIF]

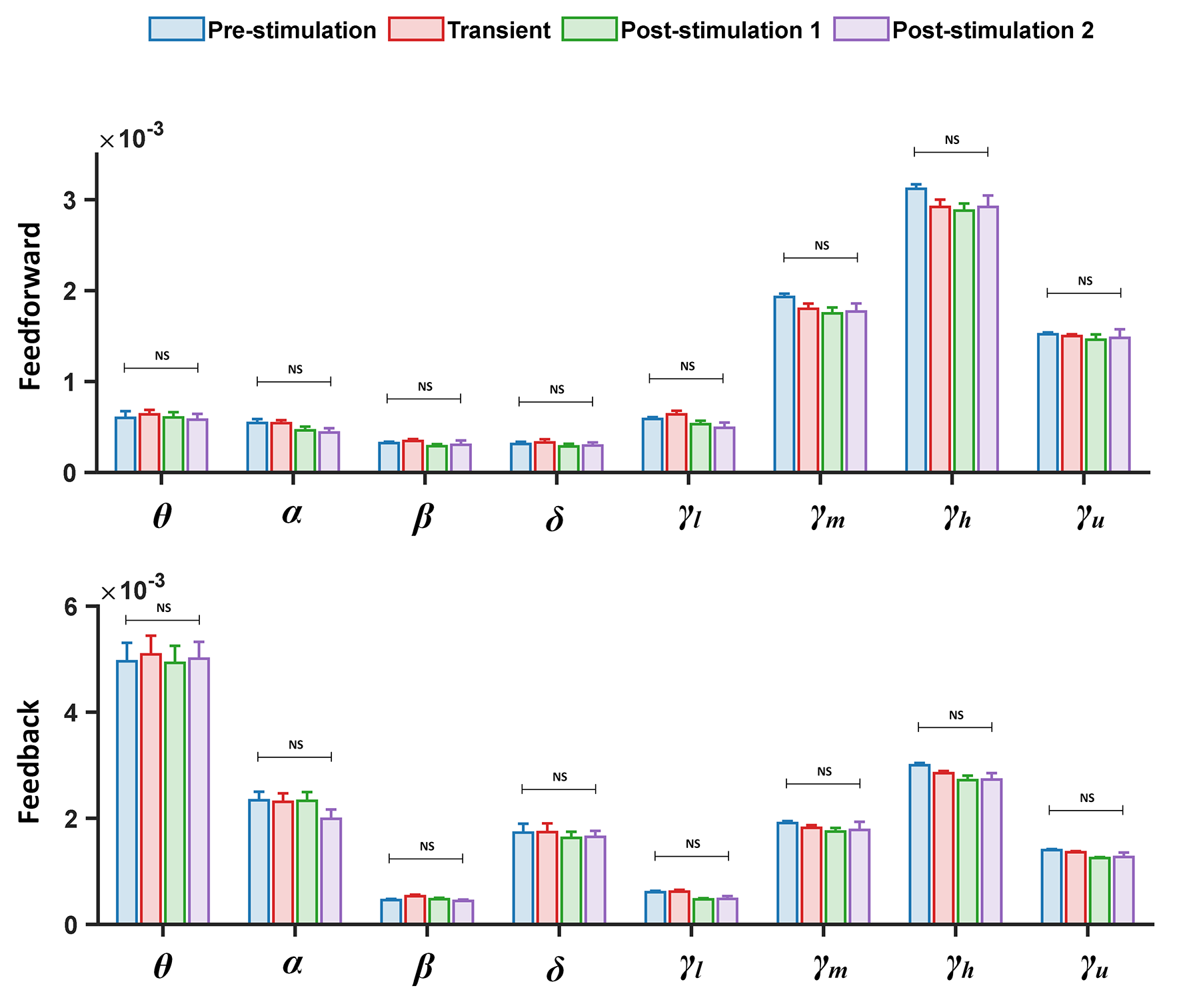

Supplement: Supplementary Figure 6 — Corticocortical directional connectivity across all analyzed bands remains unchanged in sham control rd10 mice (n = 6). The average feedforward (upper) and feedback (lower) directional connectivity across all analyzed frequency bands from the sham control group was computed during pre-stimulation (baseline), transient stimulation, post-stimulation stage 1 and post-stimulation stage 2. θ Theta; α alpha; β beta; γl low-gamma; γm medium-gamma; γh high-gamma; γu ultra-gamma; Feedforward (left primary visual cortex to left prefrontal cortex) and feedback (left prefrontal cortex to left primary visual cortex). NS, not significant; Error bar denotes SEM. [file Image_6.TIF]

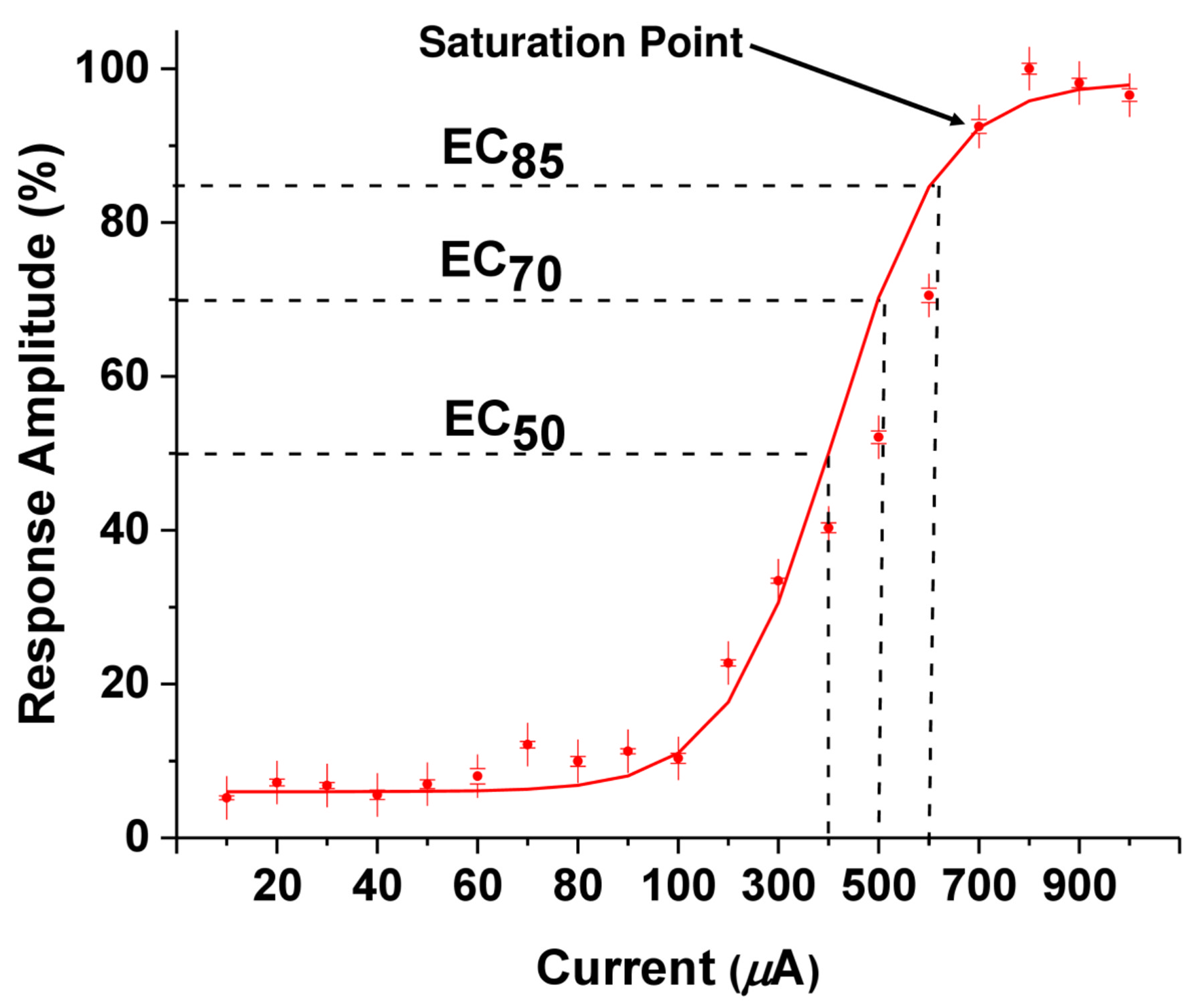

Supplement: Supplementary Figure 7 — Average dose response plot from the left primary visual cortex. The average response amplitudes were obtained from six rd10 mice. [file Image_7.TIF]
